# Phonological Feature Posteriors and Cue-Specific Accent Perception in Hindi- and Tamil-Accented English

**DOI:** 10.3390/brainsci16020177

**Published:** 2026-01-31

**Authors:** Nitin Venkateswaran, Ratree Wayland

**Affiliations:** Department of Linguistics, Turlington Hall, University of Florida, P.O. Box 115454, Gainesville, FL 32611-5454, USA; venkateswaran.n@ufl.edu

**Keywords:** accented speech, phonological features, phonetic gradience, cue-specific change, bilingual speech, neural networks

## Abstract

**Background/Objectives**: Accented speech reflects systematic deviation from target-language phonetic norms. This study demonstrates that perceived accent strength covaries with selective, gradient differences in phonological feature realization. We examine whether perceived accents in Hindi- and Tamil-accented English reflect uniform segmental deviation or cue-specific patterns of phonological feature realization. **Methods**: English speech produced by native speakers of Hindi and Tamil was evaluated using native listener accentedness ratings. Phonetic variation was analyzed using posterior probabilities of phonological features derived from a machine learning model, Phonet. The analyses focused on liquids (laterals and rhotics (e.g., /l/, /ɭ/, and /ɻ/) and labial segments in the fricative–glide space (e.g., /v/, /w/, and /ʋ/), with attention to word position and feature-level generalization. **Results**: Accentedness ratings differed systematically for Hindi- and Tamil-accented English and covaried with a subset of phonological feature dimensions, yielding contrast- and context-specific patterns of perceptually relevant variation. Not all features that varied in production contributed to perceived accent strength. **Conclusions**: These findings support a cue-specific, perception-grounded account of accentedness and establish phonological feature posteriors derived from Phonet as interpretable phonological categories through which gradient L2 production differences are evaluated by listeners.

## 1. Introduction

English, as a global lingua franca, is spoken with a wide range of accents influenced by speakers’ native languages. Indian English, a well-established variety, exhibits phonetic and phonological features shaped by the substrate influence of Indo-Aryan and Dravidian languages [1]. Within this variety, Hindi-accented English stands out due to the phonetic characteristics transferred from Hindi, a language spoken by over 500 million people. These influences contribute to systematic deviations from native English norms, affecting intelligibility, communication efficiency, and social perceptions of accentedness.

The study of Hindi-accented English has primarily focused on segmental deviations from standard American and British English pronunciations. Research has documented specific phonetic modifications, such as the realization of the English alveolar stops /t/and /d/ as retroflex [ʈ] and [ɖ], the substitution of the approximant /ɹ/ with a tap [ɾ], and the production of /w/ as a labiodental approximant /ʋ/ [2,3]. These deviations are not merely random errors but reflect the phonological constraints of Hindi, in which certain English phonemes do not have direct equivalents.

Understanding the phonetic characteristics of Hindi-accented English is crucial for improving automatic speech recognition (ASR) systems, refining models of second-language speech learning, and addressing biases in accent perception. While prior work has identified individual segmental deviations, less is known about how such deviations pattern across features and contexts, or which dimensions of variation are most relevant to listeners’ perceptions of accentedness. Recent advances in computational phonetics provide new tools for addressing these questions, enabling systematic, quantitative analyses of segmental variation beyond isolated acoustic correlates [4].

The present study examines how gradient phonological feature realization in Hindi- and Tamil-accented English relates to perceived foreign accentedness, using phonological posterior probabilities to link segmental production patterns to listener judgments. Rather than focusing on individual acoustic correlates of accentedness in isolation, we adopt a feature-based approach that examines how patterned phonological feature realization across segments and contexts covaries with perceived accent strength. Focusing on laterals and labial segments in Hindi- and Tamil-accented English, we use phonological posterior probabilities derived from Phonet [4] to quantify gradient feature realization and relate it to listener ratings of accentedness. By comparing two typologically distinct L1s within a common L2, the study aims to clarify how accentedness is associated with systematic patterns of phonological feature realization across languages and word positions.

## 2. Theoretical Framework and Language Background

### 2.1. Cross-Language Speech Perception and Accentedness

Prominent models of cross-language speech perception and learning have been developed to account for how listeners perceive and evaluate non-native speech sounds relative to the sound system of their first language [5].

The Perceptual Assimilation Model (PAM) [6,7] is grounded in a direct-realist framework [8,9], according to which perception is direct and the primary objects of speech perception are articulatory gestures rather than acoustic cues or abstract symbolic representations. Within this framework, non-native sounds are interpreted in terms of their similarity to native-language articulatory gestures and the contrastive organization of those gestures. Perceptual assimilation patterns, therefore, reflect how non-native speech tokens map onto existing gestural categories, rather than how such tokens would map onto abstract phonological feature representations.

In contrast, the Speech Learning Model (SLM) and its revised formulation (SLM-r) emphasize perceived cross-language dissimilarity as a key factor promoting the formation of new L2 categories [10,11]. SLM does not specify a single object of perception underlying cross-language mapping, focusing instead on the consequences of perceived similarity and dissimilarity for phonetic category formation and change over time. Importantly, SLM explicitly characterizes cross-language comparison as operating over position-sensitive allophones, such that an L2 segment produced in a given phonological context is evaluated relative to native productions of the corresponding segment in that same context.

Despite these theoretical differences, both frameworks share an important practical consequence: cross-language perception is treated as a segment-in-context comparison grounded in surface realizations of native and non-native speech. In empirical work, this has typically led to analyses that quantify similarity or dissimilarity using acoustic correlates diagnostic of particular segments, such as voice onset time for stop voicing or formant structure for vowels. Such measures have proven highly informative and can capture gradient variation within categories. At the same time, individual acoustic cues necessarily index only specific aspects of segmental realization and may not fully capture coordinated patterns of variation that extend across multiple properties or across natural classes of segments.

The present study adopts a segmental approach in which phonological features are treated as the basic units of representation, while allowing their realization in speech to be gradient. Thus, the analytic question is not only whether a segment is classified as a lateral (e.g., [l]), but how strongly it instantiates feature-defined properties such as [lateral] and [back]—for example, whether a given [l] token exhibits weaker lateral evidence or reduced backing relative to other tokens or contexts. We operationalize this dual categorical–gradient representation using posterior probabilities derived from Phonet. Because Phonet is trained on Mel-energy representations across broad classes of segments, its posterior estimates both classify segments with respect to feature-defined categories and quantify the strength of evidence for those features in individual tokens. This feature-based representation allows us to characterize gradient patterns of segmental realization across word positions and L1 backgrounds and to examine how such patterns covary with native listeners’ accentedness ratings.

In the following section, we describe the phonological feature framework adopted in this study and introduce the use of phonological posterior probabilities as a means of operationalizing gradient feature realization across segment classes.

### 2.2. Phonological Features and Accentedness

Phonological sound systems are traditionally modeled as structured inventories of segments organized into natural classes defined by shared features. In this framework, segments are represented as bundles of distinctive features that encode contrastive articulatory and acoustic properties, allowing phonological generalizations to be applied to classes of sounds rather than individual segments [12,13]. For example, features related to place of articulation distinguish sounds produced with different tongue configurations along the anterior–posterior dimension of the oral cavity, indexed by features such as [anterior] and [back], while manner features encode differences in constriction degree and patterns of oral airflow, such as [continuant], [sonorant], [approximant], and [lateral]. The feature framework assumed here follows the inventory proposed by [14].

In classical generative phonology, such features are typically specified categorically, often as binary values, with contrasts determined by discrete feature specifications. At the same time, distinctive features are grounded in the articulatory and acoustic properties of sounds, linking phonological structure to the phonetic signal [12]. This dual commitment—categorical organization paired with phonetic grounding—raises the question of how phonological features are realized and evaluated when their acoustic correlates vary continuously across contexts, speakers, and languages. If phonological features are instantiated via constellations of acoustic and articulatory cues, one important question is whether all cues associated with a feature change in concert over time, or whether phonetic reorganization instead proceeds in a cue-specific and selectively weighted manner.

Phonological features also provide a compact way to characterize how specific segments pattern by shared articulatory properties and how they contrast along theoretically motivated dimensions. In the present study, we focus on two sets of segmental contrasts that are well-suited for examining feature-level gradience and cue-specific change. First, for laterals and rhotics, we compare the expected American English alveolar lateral /l/ and its velarized ‘dark’ allophone [ɫ] with retroflex realizations in Hindi- and Tamil-accented English, including the retroflex lateral [ɭ] and the retroflex approximant /ɻ/. The feature dimensions implicated in these contrasts include [anterior], [back], and [lateral], reflecting differences in coronal place of articulation, tongue-body retraction or velarization, and the presence versus absence of lateral airflow. We use [back] (rather than [dorsal]) in this contrast set because variation in English /l/—including the dark [ɫ] allophone—primarily involves tongue-body retraction/velarization, which is more directly captured along a [back] dimension than by the broader major-place feature [dorsal].

Second, for the labial fricative–glide space, we compare the expected labiodental fricative /v/ and labiovelar approximant /w/ with the labiodental approximant /ʋ/ attested in Hindi- and Tamil-accented English. The relevant feature dimensions for these contrasts include [consonantal], [sonorant], [approximant], [round], [labiodental], and [dorsal], which jointly distinguish fricative and approximant manner as well as labiodental versus labiovelar place properties. These contrasts therefore provide a concrete testing ground for examining whether shared phonological features are realized uniformly across cues or whether phonetic drift manifests as cue-specific reorganization at the feature level, setting the stage for the discussion in the following section.

### 2.3. Cue-Specific Drift and Feature Weighting

While phonological features provide a structured representational framework for characterizing speech contrasts, perceptual evaluation of speech does not require uniform sensitivity to all feature dimensions. A central assumption of cue-integration approaches to speech perception is that phonological contrasts are supported by multiple acoustic dimensions, and listeners can assign different weights to these dimensions depending on language experience and structural constraints [15,16]. Under this view, phonological features and contrasts are instantiated not by single invariant cues, but by constellations of partially redundant acoustic and articulatory properties. Classic work on stop voicing, for example, has shown that contrasts are supported by numerous correlates rather than a single cue [17,18], illustrating how phonological distinctions are realized through multiple dimensions of the speech signal.

This motivates a cue-specific account of accentedness, in which accent-related differences reflect selective variation in individual phonetic dimensions contributing to a contrast, rather than categorical differences at the level of the segment. In production, speakers may maintain stable phonological contrasts while adjusting the relative strength, timing, or reliability of particular cues. Experimental work has shown that cue weights can be modified through experience and training, including selective changes in how listeners use cues to consonant voicing [19,20]. Importantly, cue weighting is also variable across individuals: listeners can differ in which cue they treat as primary, and these initial strategies predict how they adapt when the distribution of cues in the input changes [21]. In this sense, cue weighting is not fixed, but dynamically adjustable across listeners and learning contexts.

Cue-specific drift is especially relevant in bilingual speech, where the cue structure of the first language may not align with that of the second language. Models of second language speech learning emphasize that category formation and reorganization depend on perceived cross-language similarity/dissimilarity and on the learner’s ability to restructure phonetic representations across the lifespan [10,11]. Empirical evidence further shows that even relatively limited L2 experience can induce changes in L1 production, which is consistent with drift emerging through incremental reorganization rather than abrupt categorical replacement [22].

At the same time, cue-specificity is inherently relational, involving both how cues are realized in production and how they are evaluated in perception. Phonetic variation in production does not, by itself, determine perceptual relevance: some dimensions may vary robustly without contributing to listeners’ judgments, while others may exert disproportionate influence on perceived category membership or accentedness. In the present study, cue-specificity is, therefore, operationalized on the perception side, with a phonological feature treated as accent-relevant only if variation in that feature statistically predicts listeners’ accentedness judgments.

Phonet [4] is implemented as a gated recurrent unit (GRU; [23])-based neural net-work architecture designed to estimate posterior probabilities of phonological features from the speech signal. The input signal is divided into half-second windows, within which log energy is computed along the Mel scale using 33 triangular filters for each 25 ms frame. These log-energy sequences are processed by two bidirectional GRU layers, allowing the model to incorporate contextual information from both preceding and following frames. A time-distributed dense layer then estimates frame-level probabilities for each phonological feature via binary classification of feature presence versus absence. In a multi-task learning setup, separate dense layers are used for each feature, and frame-level posterior probabilities are averaged to yield a segment-level vector of feature activation. Because contextual information is integrated across time, this architecture naturally captures coarticulatory effects arising from neighboring segments [4].

### 2.4. Phonological Features Posteriors and the Phonet Model

Phonological feature posteriors provide a principled way to model the relationship between continuous phonetic variation and structured phonological representations. Because phonological contrasts are supported by multiple acoustic dimensions the relative weighting of which can vary with experience, changes in speech production may be reflected not only in individual acoustic measures but also in how strongly the speech signal supports particular phonological dimensions over time.

Phonet operationalizes this idea by using neural network models trained to estimate posterior probabilities of phonological features from acoustic representations of the speech signal (log-Mel energy features computed over short-time frames), yielding continuous and temporally resolved indices of feature evidence that can be aggregated over segments. Rather than treating features as categorical labels derived via discrete thresholding after acoustic measurement, Phonet represents feature evidence probabilistically, providing a gradient yet linguistically interpretable representation [4,24,25]. This representation is therefore well-suited for capturing distributed, sub-phonemic variation that may not be detectable through individual acoustic measures alone.

### 2.5. The Linguistic Landscape of India

India is home to one of the most linguistically diverse populations in the world, with over 1300 languages spoken across its regions [26]. The country’s official languages, Hindi and English, serve distinct yet interconnected roles: Hindi is the most widely spoken language and functions as a lingua franca across northern and central India, while English is used extensively in education, administration, and international communication [2,27]. English, introduced during British colonial rule, remains a prestige language, facilitating socioeconomic mobility and access to global networks [28].

The interaction between Hindi and English has led to a unique variety known as Indian English, which itself exhibits significant phonetic variation due to the influence of different regional languages [1]. Within this spectrum, Hindi-accented English is one of the most widely studied varieties, given the large number of Hindi speakers who use English as a second language [29]. The phonetic influence of Hindi on English pronunciation results in systematic deviations from native English norms, shaping perceptions of accentedness and intelligibility in multilingual contexts.

This paper examines the phonetic characteristics of Hindi- and Tamil-accented English, comparing them with American English, and investigates how these differences shape perceptions of foreign-accented speech.

### 2.6. Phonological Characteristics of Hindi vs. English

Hindi and English, though both Indo-European languages, differ significantly in their phonological systems, leading to systematic adaptations in Hindi-accented English. Table 1 presents a comparison of Hindi and English consonant phonemes, while Table 2 compares their vowel phonemes.

The Hindi phonemic inventory is based on standard Hindi, as described by [30]. Hindi’s consonantal system reflects Indo-Aryan phonological characteristics and is shaped by influences from loanwords in Persian, Arabic, English, and Sanskrit. It has a rich consonantal inventory that includes plosives, affricates, nasals, taps/flaps, fricatives, approximants, and laterals, distributed across bilabial, labiodental, dental, alveolar, retroflex, post-alveolar, palatal, velar, and glottal places of articulation.

One of the defining features of Hindi’s phonological system is its four-way stop contrast, which distinguishes between voiceless, voiced, aspirated, and breathy-voiced (voiced aspirated) stops. Specifically, Hindi contrasts /p, p^h^, b, b^ɦ^/, whereas English lacks a phonemic distinction between /b/ and /b^ɦ^/ or /p/ and /p^h^/. In English, aspiration serves as an important cue for differentiating voiceless stops (e.g., [p^h^] in ‘pat’ vs. [p] in ‘spat’), but it is not phonemically contrastive, as it is in Hindi.

Hindi also has a distinct set of retroflex sounds, including stops /ʈ, ʈ^h^, ɖ, ɖ^ɦ^/ and flaps /ɽ, ɽ^ɦ^/, none of which exist in English. Additionally, Hindi features an alveolar tap /ɾ/ instead of the alveolar approximant /ɹ/ found in English, which further contributes to its phonological distinctiveness.

Certain consonants in Hindi originate from borrowings and historical developments. The fricatives /f, z, ʒ/ are found mainly in loanwords from Persian, Arabic, and English but are now well established in Modern Standard Hindi. Some sources also include /ɭ/ (a retroflex lateral), which appears in a few Sanskrit borrowings, though it is often replaced by /n/ in casual speech. Additionally, the sounds /x, ɣ, q/ do not occur in the variety of Hindi described by Ohala, but are present in Urdu, the standardized register of Hindustani used as Pakistan’s official language and widely spoken by Indian Muslims.

Ohala [30] notes that all Hindi consonants can occur as geminates, except /b^ɦ^/, the rhotic flap, /w/, and /h/. Geminates appear only medially, always following /i, a, u/, and are typically twice as long as their singleton counterparts. While orthography retains some word-final geminates, they are usually pronounced as singletons except in formal speech. Most geminates occur mono-morphemically, except for /dʒː/, which appears in Sanskrit loans where a morpheme boundary can be posited.

According to Ohala [30], Hindi has eleven oral vowels, with [æ] appearing only in English loanwords. All of these vowels except [æ] have distinct nasalized counterparts. Additionally, the vowel sequences /əɪ/ and /əʊ/ occur in Hindi but are analyzed as vowel clusters rather than diphthongs. These phonetic and phonological differences shape Hindi-accented English, influencing both intelligibility and listener perception. Understanding these systematic variations is crucial for research in second-language acquisition, speech recognition, and sociophonetics, as well as for effective communication in multilingual settings.

### 2.7. Phonological Characteristics of Hindi vs. Tamil

Table 3 compares the Hindi and Tamil consonant inventories (Hindi: [30]; Tamil: [31]; Central Tamil/Madurai: [3]). Both languages share three coronal places—dental, alveolar, and retroflex—and each maintains a robust retroflex stop series. They diverge in several core respects. Hindi exhibits a four-way stop contrast with phonemic voicing and aspiration across places (e.g., /p, p^h^, b, b^ɦ^/), whereas Tamil lacks phonemic voicing and aspiration contrasts in native stops; Tamil allophonic stops are typically voiceless (often weakly aspirated) word-initially and voiced after nasals [3,32]. Hindi also has a richer affricate and fricative set (including /tʃ, tʃ^h^, dʒ, dʒ^ɦ^/ and /f, s, z, h), while Central Tamil only has /tʃ/ as the primary affricate and only /s, ʂ/ among fricatives. According to [3], Tamil voiced retroflex approximant is slightly fricativized, /ɻ̝/.

In the liquids/rhotics, Tamil uniquely contrasts a retroflex lateral /ɭ/ and retroflex /ɻ̝/, whereas Hindi has a retroflex flap /ɽ/ but lacks phonemic /ɽ/ and /ɭ/. At the labial place, both systems include a labial approximant /ʋ/, but Hindi also contrasts a labiodental fricative /f/ [30].

As for the vowel systems (Table 4), Tamil has a five-vowel system with contrastive length—ten underlying vowels overall—with additional quality differences in the short vs. long high vowels. Tamil /i, e, o, u/ have lax counterparts [ɪ, ɛ, ʊ, ɨ] in non-initial syllables [31]. Hindi has a larger oral vowel inventory that contrasts primarily in quality (including phonemic /ə/, realized as [ɐ]), with [æ] limited to English loanwords; all oral vowels except [æ] have phonemic nasalized counterparts [30].

### 2.8. Phonological Characteristics of English vs. Tamil

Table 5 compares English and Tamil consonant inventories (Tamil: [31]; Central Tamil/Madurai adjustments: [3]). Both languages share bilabial, alveolar, palatal, and velar places and include a labial approximant. They diverge in several core respects: English contrasts voicing across plosive places (/p, b, t, d, k, ɡ/) and uses aspiration allophonically, and has a richer fricative set (/f, v, θ, ð, s, z, ʃ, ʒ, h/) and an alveolar approximant /ɹ/ with contextually dark [ɫ]. By contrast, Tamil lacks phonemic voicing/aspiration in native stops and has a more limited fricative inventory. Tamil also maintains a robust retroflex series and employs tap /ɾ/, categories not matched phonemically in English. In the labial space Tamil uses /ʋ/ rather than a /v/–/w/ contrast [3,31].

Table 6 compares the English and Tamil vowel systems. As mentioned earlier, Tamil has five vowel qualities with contrastive length, yielding ten underlying vowels; the short–long high pairs also differ in quality. Importantly, in non-initial syllables, /i, e, o, u/ show non-contrastive lax allophones [ɪ, ɛ, ɔ, ʊ], and nasalization is non-phonemic and largely restricted to word-final position [3,31]. English, by contrast, has a larger oral vowel inventory with multiple contrastive tense–lax pairs as well as three diphthongs (/aɪ, aʊ, ɔɪ/). These structural differences—length-driven contrasts in Tamil versus quality and diphthong-based contrasts in English—are potential sources of common L1 → L2 vowel mappings in Tamil-accented English.

## 3. Materials and Methods

This section provides an overview of datasets used to train Phonet to generate phono- logical feature probabilities, the CSLU Foreign Accented English dataset [33] containing the spoken English of native Hindi and Tamil speakers, and the analysis procedures used to determine gradient phonological feature differences across accented speaker groups.

### 3.1. Contrastive Features Investigated

We investigate the phonological features that are contrastive among the lateral segments of American English, Hindi-accented English, and Tamil-accented English, which are listed in Table 7.

We also investigate the contrastive features of the labiodental approximant /ʋ/ productions in Hindi English and Tamil English where the labial fricative /v/ or labiovelar approximant /w/ realizations in American English would be expected; these are listed in Table 8.

### 3.2. Datasets

To train Phonet models to generate posterior probabilities for segments, we use large speech corpora of American English, Hindi English, and Tamil English, along with their transcriptions, from the Mozilla Common Voice project [35], the Librispeech-100 corpus [36], the L2-ARCTIC corpus of non-native English speech [37], the Indic Text-To-Speech corpus [38] and the IndicTIMIT corpus [39].

For analysis, we use the CSLU Foreign Accented English (FAE) Release 1.2 dataset [33] consisting of spontaneous speech in English by native speakers of 22 languages, along with accent annotations for each speech utterance. We focus on the subset of utterances produced by native Hindi and Tamil speakers. The utterances are telephone-quality, with speakers asked to speak about themselves in English for 20 s. Each speaker produces only one recording, for a total of 349 recordings by native Hindi speakers and 326 recordings by native Tamil speakers.

**Figure 1:**
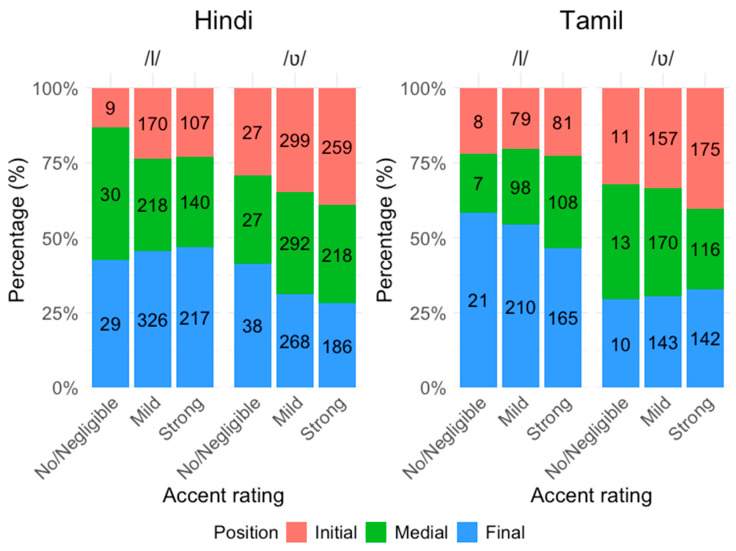
Distribution of lateral /l/ and labiodental approximant /ʋ/ segments produced by native Hindi and Tamil speakers in the CSLU FAE dataset. Counts are grouped by accent rating and word position.

Three native speakers of American English rated each utterance for accent strength on a 4-point scale: 1—negligible/no accent, 2—mild accent, 3—strong accent and 4—very strong accent. To establish a conservative estimate of perceived accent strength, we adopt the average rating across the three raters as the final score for each recording, and we merge the ‘very strong’ category with the ‘strong’ category, given the low resulting frequencies (<1%) of the ‘very strong’ rating. This results in three final levels: no/negligible, mild, and strong. Figure 1 shows the distribution of the lateral /l/ and labiodental approximant /ʋ/ segments by accent rating and word position, in the recordings produced by the native Hindi and Tamil speakers.

### 3.3. Montreal Forced Aligner (MFA) Pre-Processing

The Montreal Forced Aligner (MFA) [40] is used to force-align the audio and phone segments for all datasets, with the resulting TextGrid files being used to annotate the phonological feature classes associated with each audio frame via the frame’s aligned phone segment. The audio transcripts are transcribed into IPA segments using the pre-trained MFA grapheme-to-phoneme (G2P) models and pronunciation dictionaries [41,42,43,44]. Custom acoustic models for American English, Hindi English, and Tamil English are trained to avoid potentially noisy output from the existing pre-trained acoustic model [45], given that this pre-trained model is trained on a variety of forms of English worldwide. Training follows the standard four-stage pipeline used to train acoustic models in MFA, i.e., monophone model training followed by triphone model training, LDA+MLLT feature extraction and MFCC-based speaker adaptation.

### 3.4. Phonet Training Procedure

Two Phonet models are trained: one on a combined American English and Hindi-accented English training set, and another on a combined American English and Tamil-accented English training set. This joint training allows each model to learn probabilistic mappings between acoustic representations and phonological features informed by both varieties, ensuring that differences across varieties reflect differences in phonological feature realization rather than artifacts of separate model training. The probabilistic mappings are trained using a table mapping the segments in acoustic representations to their phonological features, which is detailed in Table A3 in Appendix A. While [retroflex] has been proposed as a distinctive feature in some accounts, the feature inventory adopted here ([14], pp. 95–97) does not include [retroflex] as a distinct feature; therefore, it is not represented in the implemented phone–feature mapping. Accordingly, retroflex-related place differences relevant to the present contrasts are examined using the available coronal place feature [anterior] as a partial proxy for posterior coronal place. For segments in the Hindi- or Tamil-accented English data from the CSLU FAE corpus, the corresponding Phonet model estimates graded phonological feature evidence that can be interpreted relative to distributions observed in American English and in the respective accented English data, making it possible to assess whether feature realizations pattern more closely with American English or with the accented variety, or whether they show intermediate behavior without assuming categorical distinctions between native and non-native speech.

Phonet probabilities, produced for every 10 ms frame in the speech signal, are averaged across the middle set of frames of the segment, with the initial and final frames of the segment excluded. An 80-20 train–test split is used for training the Phonet models, with separate binary classification heads used to generate feature probabilities for each phonological feature of the segment in a multi-task setup. The range of accuracy and F1 scores across the phonological classes can be found in Table A1 and Table A2 in Appendix A. The model is trained for a maximum of 30 epochs with early stopping, using the Adam optimizer [46] with a categorical cross-entropy loss function.

### 3.5. Statistical Analyses

Mixed-effects models are run using the lmerTest package version 3.1.3 [47] in R version 4.4.1 [48], with the posterior probability of the phonological feature as the dependent variable; the speaker and IPA transcription of the segment’s word context as random effects; and the accent rating, word position of the segment, and the interaction of accent rating and word position as fixed effects. Models are developed for each English variety (Hindi and Tamil English) and feature combination, i.e., for the [anterior], [lateral], and [back] feature probabilities when analyzing the lateral segments, and for each of the contrastive features between the ʋ/v and ʋ/w pairs (i.e., [sonorant], [approximant], and [consonantal] for the ʋ/v pair and [round], [labiodental], [dorsal], [back], [high] and [tense] for the ʋ/w pair). Statistical significance of the fixed effects is analyzed through likelihood ratio tests using the anova() function from the car package version 3.1.3 [49]. Post hoc analyses are conducted via pairwise *t*-tests with Bonferroni correction using the emmeans package [50], to investigate probability differences across the accent rating, word position, and interaction of accent rating and word-position groups, with degrees of freedom being approximated using the Satterthwaite method [51].

## 4. Results

The results examine which dimensions of phonological feature realization are associated with perceived accent strength, and whether these relationships are conditioned by word position and L1 background. Table 9 and Table 10 summarize the statistically significant pairwise differences in phonological feature probabilities by accent rating, word position, and their interaction, separately described for Hindi- and Tamil-accented English.

For Hindi-accented English (Table 9), accent-related effects are limited to a subset of feature dimensions. While [anterior] and [lateral] probabilities vary robustly by word position, only the variation in [back] probabilities is systematically associated with accent ratings for lateral segments, with stronger accented speech being associated with reduced [back] probabilities. In the labial domain, stronger accent ratings are associated with increased [sonorant] and [approximant] evidence in productions of expected /v/, with effects that are strongest in non-initial positions.

In contrast, a more context-dependent pattern is evident for Tamil-accented English (Table 10). Reduced [back] probabilities in lateral segments are again associated with stronger accent ratings, but effects involving [lateral] evidence emerge only in word-final position. Unlike Hindi speakers, Tamil speakers show no systematic accent-related effects for productions of expected /v/, while accent ratings for /w/ productions are associated with reduced evidence for tongue-body-related features.

The following sections unpack these patterns with statistical details.

### 4.1. Lateral Segments

**Figure 2:**
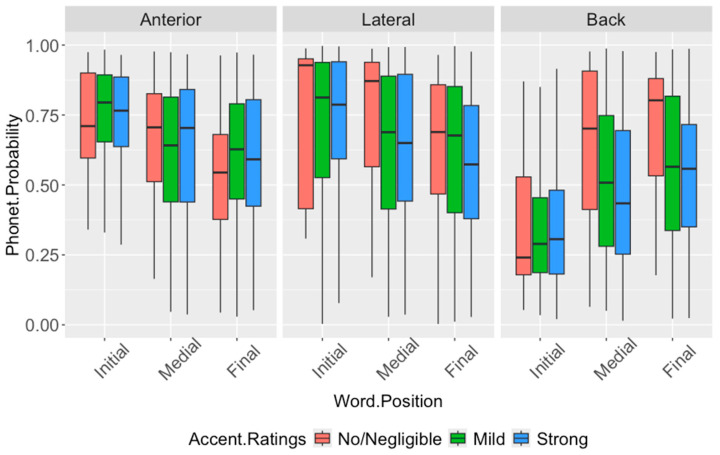
Distribution of [anterior], [back], and [lateral] probabilities of lateral segments in the English of native Hindi speakers, by word position and accent rating. Word-medial and word-final laterals show significantly lower [anterior] and [lateral] probabilities than word-initial laterals. As for [back] probabilities, mild and strong accents show significantly lower probabilities relative to the no/negligible accent. Significant pairwise comparisons (Bonferroni-corrected) are summarized in Table 9.

We first examine lateral segments, focusing on which aspects of lateral feature realization are associated with perceived accent strength across Hindi- and Tamil-accented English. Lateral productions in the English of native Hindi and Tamil speakers are evaluated relative to expected productions of /l/ in American English, including the ’light’ [l] and ‘dark’ [ɫ] allophones. Figure 2 shows the probability distributions of the [anterior], [lateral], and [back] features in Hindi English lateral segments, by accent rating and word position.

Looking at productions by native Hindi speakers, the results show significant main effects of word position on the probabilities of all classes (anterior: F_(2,327)_ = 15.08, *p* < 0.0001; lateral: F_(2,299)_ = 10.13, *p* < 0.0001; back: F_(2,388)_ = 27.64, *p* < 0.0001). There are no significant effects of accent rating on the [anterior] and [lateral] class probabilities (anterior: *p* = 0.73; lateral: *p* = 0.30); however, the effect of accent rating on [back] probabilities is significant (back: F_(2,255)_ = 6.07, *p* = 0.0026).

Pairwise *t*-tests show significantly lower [anterior] and [lateral] probabilities, as well as higher [back] probabilities word-medially (anterior: *p* = 0.0058; lateral: *p* = 0.013; back: *p* = 0.0004) and word-finally (anterior, lateral, back: *p* < 0.0001) relative to word-initial; [anterior] and [back] probabilities are also significantly different word-finally vs. word-medially (anterior: *p* = 0.047; back: *p* = 0.0009). The mild and strong accents show significantly lower [back] probabilities than does the no/negligible accent (mild: *p* = 0.0077; strong: *p* = 0.0018). These results suggest that the degree of anteriority and lateral airflow does not impact the strength of the accent perceived, though these vary by word position, with less prominent anteriority and lateral properties word-medially and word-finally. In addition, while native Hindi speakers may be producing laterals word-medially and word-finally using articulators further back in the mouth, the degree of backness may still be perceptibly lower than that found in the dark-l variant [ɫ] of American English in the corresponding positions, with the differences in perception contributing to accent strength.

**Figure 3:**
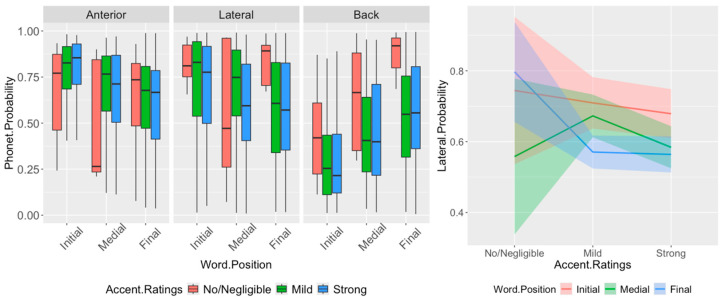
(**Left**): Distribution of [anterior], [back], and [lateral] probabilities of lateral segments in the English of native Tamil speakers, by word position and accent rating. [back] probabilities differ significantly across all word positions (final > medial > initial) and are significantly lower for mild and strong accents than for the no/negligible accent condition. [lateral] probabilities show a significant accent-related reduction specifically in word-final position (mild/strong < no/negligible). (**Right**): Interaction plot of [lateral] probabilities by accent rating and word position (with confidence intervals), highlighting the word-final-specific [lateral] effect. Significant Bonferroni-corrected pairwise comparisons are reported in Table 10.

Figure 3 shows the probability distributions of the [anterior], [lateral] and [back] features in Tamil English lateral segments, shown by accent rating and word position. The results show significant effects of word position (F_(2,336)_ = 19.41, *p* < 0.0001) and accent rating (F_(2,201)_ = 9.19, *p* = 0.00015) on the [back] probabilities. Pairwise *t*-tests show significant differences in [back] probabilities across all word-position pairs, with final > medial > initial probabilities, as well as significantly lower probabilities for strong (*p* = 0.0002) and mild (*p* = 0.0001) accents compared to the no/negligible accent. Interaction effects between accent rating and word position on the [anterior] and [lateral] probabilities are significant (anterior: F_(4,725)_ = 2.43, *p* = 0.046; lateral: F_(4,737)_ = 19.41, *p* = 0.035), and the main effect of word position on [anterior] probabilities is also significant (F_(2,620)_ = 4.33, *p* = 0.013), though the effect of accent rating is not (*p* = 0.15). Pairwise *t*-tests show significantly higher word-initial [anterior] probabilities relative to word-final for the mild and strong accents (initial vs. final|mild: *p* = 0.0001; initial vs. final|strong: *p* < 0.0001), and significantly higher initial than medial [anterior] probabilities for the strong accent (initial vs. medial|strong: *p* = 0.049). Analyses of interaction effects by accent rating for the [lateral] case also show higher word-initial and word-medial probabilities as compared to word-final for the mild accent (initial vs. final|mild: *p* = 0.0056; medial vs. final|mild: *p* = 0.033), and higher word-initial than word-final probabilities for the strong accent (initial vs. final|strong: *p* = 0.039).

Looking at interaction effects by word position for the [anterior] case, the analyses show no significant pairwise effects for any accent rating pairs. In the [lateral] case, probabilities are lower for the strong and mild accents only in word-final position (no/negligible vs. mild|word-final: *p* = 0.022; no/negligible vs. strong|word-final: *p* = 0.018). The results overall suggest that, unlike for Hindi English, the degree of lateral airflow influences accent perception in the word-final position; lower [lateral] probabilities are associated with stronger accent ratings. While the degree of anteriority does not affect accent perception, the anteriority is still significantly less prominent in word-final position. As with the native Hindi speakers, a lower degree of backness in lateral segments is significantly associated with a stronger perceived accent.

### 4.2. Labiodental Approximant

We compare labiodental approximant /ʋ/ productions in the English of native Hindi and Tamil speakers versus the expected /v/ and /w/ segments; this is presented in separate analyses below.

#### 4.2.1. /ʋ/ vs. /v/

**Figure 4:**
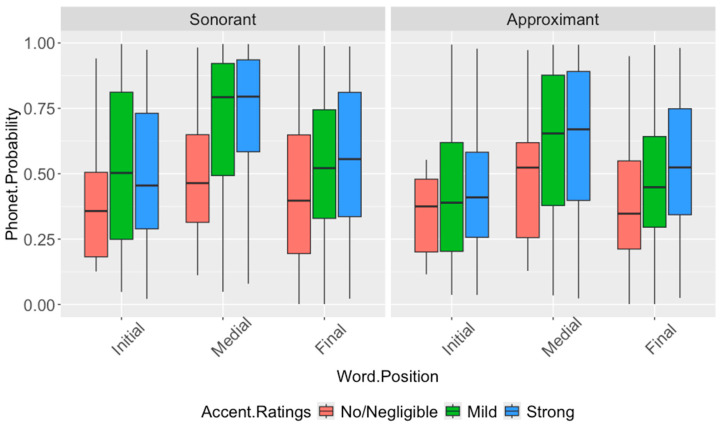
Distribution of [sonorant] and [approximant] probabilities of the expected /v/ segment in the English of native Hindi speakers, by word position and accent rating. [sonorant] probabilities are significantly higher for mild and strong accents than for the no/negligible accent condition, and [approximant] probabilities are significantly higher for the strong accent than for the no/negligible condition; the largest differences are observed in the word-medial position. Significant Bonferroni-corrected pairwise comparisons are reported in Table 9.

Figure 4 shows the probability distributions of the [sonorant] and [approximant] features, by accent rating and word position, in productions of the expected /v/ segment in Hindi English.

The results show significant effects of word position on the [sonorant] and [approximant] probabilities (sonorant: F_(2,89)_ = 12.65, *p* < 0.0001; approximant: F_(2,128)_ = 6.88, *p* = 0.0014). Pairwise *t*-tests reveal significantly higher [sonorant] probabilities word-medially than initially (*p* < 0.0001) as well as higher probabilities word-medially than word-finally (*p* = 0.0012); for the [approximant] case, word-medial probabilities are higher than word-initial (*p* = 0.0009). There are also significant effects of accent rating on the [sonorant], [approximant], and [consonantal] probabilities (sonorant: F_(2,215)_ = 5.644, *p* = 0.0049; approximant: F_(2,218)_ = 4.08, *p* = 0.018; consonantal: F_(2,203)_ = 4.91, *p* = 0.0082). Pairwise *t*-tests show higher [sonorant] probabilities for the mild (*p* = 0.012) and strong (*p* = 0.0035) accents compared to the no/negligible accent, higher [approximant] probabilities for the strong accent versus the no/negligible accent (*p* = 0.027), and higher [consonantal] probabilities for the mild accent versus the strong accent (*p* = 0.024). The results suggest that the labiodental approximant /ʋ/ is prominent in the word-medial position, and contributes to the perception of stronger accents due to stronger sonorant and approximant qualities when compared with the expected /v/ segment across all word positions.

For native Tamil speakers, the results show no significant effect of word position or accent rating on any of the contrastive [sonorant], [anterior] and [consonantal] probabilities. This suggests that, unlike the Hindi speakers, the Tamil speakers do not use the labiodental approximant /ʋ/, and produce the expected /v/ segment in their English.

#### 4.2.2. /ʋ/ vs. /w/

**Figure 5:**
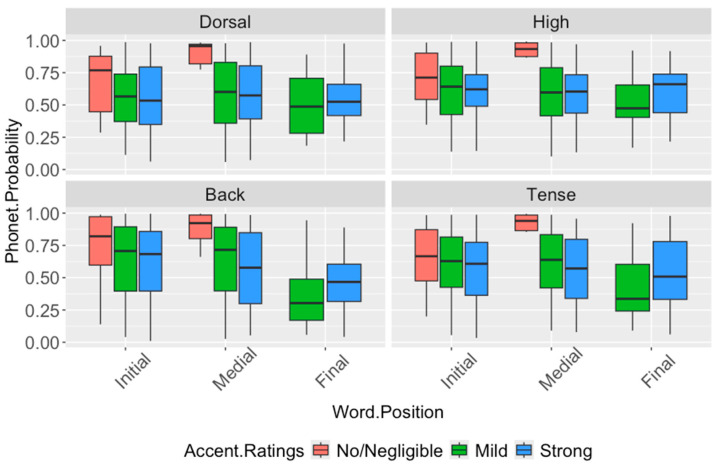
Distribution of [dorsal], [high], [back], and [tense] probabilities of the expected /w/ segment in the English of native Tamil speakers, by word position and accent rating. The no/negligible accent condition shows significantly higher probabilities than the strong accent condition for all four features, and significantly higher [high] probabilities than the mild accent condition. No significant word-position effects are observed. Significant Bonferroni-corrected pairwise comparisons are reported in Table 10.

The results for native Hindi speakers show significant effects of accent rating on [labiodental] probabilities (F_(2,225)_ = 3.34, *p* = 0.037); the average probabilities for this segment are highest for mild accents, followed by the strong and no/negligible accents. However, pairwise *t*-tests did not survive Bonferroni correction, with no significant pairwise differences being found. There are significant effects of word position on [back] probabilities (F_(2,112)_ = 3.29, *p* = 0.041), with average probabilities much higher in initial and medial positions than final; pairwise tests show that only the initial–final pair is significant (*p* = 0.034). There are no significant effects of accent rating or word position on the other features contrasting the ʋ/w pair. The results, overall, suggest that Hindi speakers do not use the labiodental approximant /ʋ/, and produce the /w/ segment in their English, and only manifest slight, gradient changes in a few articulatory features, viz., tongue backness by word position. Figure 5 shows the probability distributions of the [dorsal], [high], [back] and [tense] features by accent rating and word position, in productions of the expected /w/ segment in Tamil English.

The results show significant effects of accent rating on the [dorsal], [high], [back] and [tense] probabilities (dorsal: F_(2,121)_ = 3.09, *p* = 0.048; high: F_(2,443)_ = 4.89, *p* = 0.0078; back: F_(2,143)_ = 3.88, *p* = 0.0023; tense: F_(2,123)_ = 4.43, *p* = 0.014), with no effect of word position found for any feature. Pairwise *t*-tests reveal higher [dorsal], [high], [back], and [tense] probabilities for the no/negligible accent compared to the strong accent (dorsal: *p* = 0.044; high: *p* = 0.009; back: *p* = 0.021; tense: *p* = 0.015), and higher [high] probabilities for the no/negligible accent compared to the mild accent (*p* = 0.006). There are no significant differences of accent rating or word position on the [round] and [labiodental] features. The results, overall, show that listeners perceive an accent driven by differences in velarization of the /w/ segment and not by differences in labial articulation, with stronger accents showing less pronounced articulations with the tongue body while maintaining lip roundedness and lack of dental contact.

## 5. Discussion

The present study examined cue-specificity at the perception–production interface by asking which dimensions of phonological feature evidence are perceptually consequential for native listeners’ accentedness judgments when evaluating Hindi- and Tamil-accented English. Although accentedness has long been understood to reflect selective sensitivity to particular properties of non-native speech, the present work approaches this selectivity through a feature-based representational framework. Using posterior probabilities of phonological features, we evaluate which aspects of gradient phonetic variation covary with accent ratings and which do not. Importantly, in this study cue-specificity is operationalized on the perception side: a phonological feature is treated as accent-relevant only insofar as variation in that feature predicts listeners’ accent judgments. This framing allows us to distinguish production-side variability from perceptual relevance without presupposing categorical substitution or uniform deviation from native norms.

### 5.1. Accent Perception Reflects Selective Phonological Feature Sensitivity

A central finding of this study is that native listeners’ accent judgments are selectively sensitive to only a subset of the phonological feature dimensions that vary in production. Across both Hindi- and Tamil-accented English, several features exhibited robust variation as a function of word position and speaker group. However, only some of these dimensions systematically covaried with perceived accent strength.

For lateral segments produced by Hindi speakers, probabilities of the [anterior] feature—reflecting how far forward the coronal constriction is produced in the oral cavity— and the [lateral] feature—reflecting evidence for sustained lateral airflow—varied reliably across word positions, with lower values in the medial and final positions than in the word-initial position. Despite this systematic production variation, neither feature showed a consistent relationship with accent ratings. In contrast, [back] probabilities were significantly lower for mild and strong accents than for the no/negligible accent, indicating reduced evidence for tongue-body retraction or velarization in lateral segments judged as more strongly accented. A comparable dissociation was observed for Tamil speakers, though with a more restricted pattern of perceptual relevance. As with Hindi-accented English, reduced [back] probabilities in lateral segments—corresponding to weaker tongue-body retraction or velarization—were associated with stronger accent ratings across word positions. In contrast, variation in [anterior] evidence did not contribute to accent judgments, despite systematic positional effects in production. Additionally, reduced [lateral] probabilities—indicating less evidence for sustained lateral airflow—were associated with stronger accents specifically in word-final position, a context in a context in which /l/ shows strong positional allophony in English varieties (including coda darkening and, in some dialects, /l/-vocalization; [52]). More importantly, these patterns show that only a subset of production-side variation is perceptually consequential.

### 5.2. L1-Specific Pathways to Accentedness: Hindi vs. Tamil

Although both Hindi- and Tamil-accented English exhibited selective covariation between phonological feature evidence and accent ratings, the specific configurations of perceptually relevant features differed across the two L1 groups. These differences highlight that perceptual relevance is shaped by how feature evidence patterns across segments and contexts within a given L1–L2 pairing. For Hindi-accented English, accent ratings were consistently associated with reduced [back] evidence in lateral segments and with increased [sonorant] and [approximant] evidence in productions of expected /v/. The latter pattern indicates that productions judged as more strongly accented showed greater evidence for sonorant- and approximant-like realization in contexts where a labiodental fricative is expected, particularly in word-medial position. In contrast, variation in [anterior] and [lateral] evidence for laterals—although systematic in production—did not influence accent judgments. For Tamil-accented English, accent ratings again covaried with reduced [back] evidence in lateral segments, and, additionally, with reduced [lateral] evidence in the word-final position. In contrast to Hindi speakers, no systematic relationship between accent ratings and phonological feature evidence was observed for productions of expected /v/. These results demonstrate that cross-linguistic differences in production do not necessarily function as accent cues for all speaker groups, and that perceptual relevance is L1-specific rather than universal.

The fact that the same phonological feature dimensions contributed to accent judgments for one speaker group but not the other suggests that perceptual relevance is shaped not by feature identity alone, but by how feature evidence patterns across contexts within a given L1–L2 pairing. For both Hindi- and Tamil-accented English, reduced [back] evidence in lateral segments was associated with stronger accent ratings, which is consistent with the central role of velarization in the realization of English /l/ across positions.

In contrast, reduced [lateral] evidence contributed to accent judgments only for Tamil speakers and only in word-final position, a context in which laterality is maintained in native English and thus may be especially salient when reduced. Similarly, feature evidence associated with the /v/ contrast contributed to accent judgments for Hindi speakers but not for Tamil speakers, despite surface similarities in the speakers’ L1 inventories. These dissociations suggest that listeners are sensitive to systematic but gradient L1-specific patterns of deviation from English phonological expectations, rather than sensitive to the presence or absence of individual features in isolation.

### 5.3. Word Position as a Conditioning Factor in Accent Perception

Word position played a central role in conditioning both phonological feature realization and its relationship to accent ratings. Across analyses, lateral segments produced word-medially and word-finally exhibited lower [anterior] and [lateral] probabilities and higher [back] probabilities relative to word-initial segments. However, only some of these positional effects were associated with perceived accent strength. For Hindi speakers, positional variation was observed for all three lateral features, yet only variation in [back] evidence covaried with accent ratings. For Tamil speakers, the relationship between [lateral] evidence and accent ratings emerged exclusively in the word-final position, at which reduced laterality was associated with a stronger perceived accent. In other positions, variation in laterality did not influence listeners’ judgments. These results indicate that word position is not merely a source of phonetic variation, but a conditioning environment that determines which phonological features are perceptually relevant. Accent perception is therefore sensitive to the interaction between phonological features and the contexts in which they are realized.

### 5.4. Labial Segments and Selective Substitution Patterns

Analyses of labial segments further illustrate the contrast-specific nature of perceptual weighting in accent evaluation. For Hindi speakers, productions of expected /v/ showed increasing [sonorant] and [approximant] evidence with increasing accent strength—[sonorant] reflecting greater openness of the vocal tract with sustained voicing, and [approximant] reflecting reduced constriction and absence of turbulent air flow—particularly in the word-medial position. These feature shifts were consistently correlated with accent ratings, suggesting that deviations along these dimensions are perceptually consequential for Hindi-accented English.

On the other hand, Tamil speakers showed no systematic relationship between accent ratings and phonological feature evidence for expected /v/, despite the presence of a labial approximant in the L1 inventory. This absence of perceptual covariation underscores that production differences alone do not guarantee perceptual relevance.

For Tamil productions of expected /w/, accent ratings covaried with reduced evidence for tongue-body-related features ([dorsal], [high], [back], and [tense]), while [round] and [labiodental] evidence remained stable across accent levels. This pattern suggests that listeners’ judgments were sensitive to reduced tongue-body involvement rather than to changes in labial articulation per se. Crucially, these results suggest that perceptual weighting of labial contrasts is shaped by how deviations from English phonological expectations manifest across L1 backgrounds, rather than by the presence or absence of individual phonological features in isolation.

### 5.5. Implications for Models of Accented Speech and Phonological Representation

The present findings have several implications for how accentedness is conceptualized and studied. First, the results reinforce the view that perceived accentedness reflects selective perceptual sensitivity rather than uniform phonetic deviation. Across contrasts, only a subset of the phonological feature dimensions that varied in production contributed to listeners’ accent judgments, while other dimensions were effectively ignored. This dissociation suggests that accentedness cannot be reduced to aggregate distance from native norms, but instead emerges from how listeners weight specific dimensions of phonological evidence.

Second, the results highlight the importance of the representational level in modeling accent perception. Prior work has often examined accent perception by testing individual acoustic correlates in isolation. By contrast, the present study operationalizes perceptual selectivity using posterior probabilities of phonological features, which integrate multiple acoustic cues while preserving phonological interpretability. This feature-based approach allows perceptually relevant dimensions to be identified directly from listener judgments, without presupposing which acoustic measures should matter or how they should be combined.

Third, the observed L1-specific patterns of perceptual relevance suggest that listener sensitivity is shaped by structured expectations about how English contrasts are realized, and that these expectations interact with systematic patterns of deviation across L1 back-grounds. Features such as [back] in laterals contributed to accent judgments across groups, consistent with their central role in English contrast realization, whereas other features were perceptually relevant only in restricted contexts or for specific L1s. These findings suggest that cue-specificity in accent perception is conditioned not only by feature identity, but by how feature evidence patterns across segments and contexts relative to listener expectations.

Finally, the present results underscore the value of treating cue-specificity as a phenomenon that can be empirically defined on the perception side. Although cue-specificity is often discussed in terms of speaker-side reorganization or learning, the present study demonstrates that it can be identified through systematic covariation between phonological feature evidence and listener judgments, without assuming categorical substitution or representational change. This perception-grounded operationalization provides a principled basis for comparing accent patterns across speaker groups and contrasts, and for integrating production variability with perceptual outcomes in a unified framework.

## 6. Conclusions

This study examined cue-specificity in foreign-accented speech by asking which dimensions of phonological feature evidence are perceptually relevant for native listeners’ accentedness judgments. Across Hindi- and Tamil-accented English, listeners’ ratings covaried with only a subset of the phonological features that varied in production, demonstrating that perceived accentedness reflects selective perceptual weighting rather than uniform phonetic deviation.

The novelty of the present work lies in its use of posterior probabilities of phonological features to operationalize this selectivity in a principled and interpretable way, allowing perceptually relevant dimensions to be identified directly from listener judgments. Critically, these findings suggest that accented speech reflects systematic modification in the realization of phonological feature dimensions, whereby certain dimensions are selectively modified in production and treated as accent-relevant by native listeners. This feature-based, perception-grounded perspective provides a flexible framework for linking gradient production variability to perceptual outcomes while remaining agnostic about the precise mechanisms through which such reorganization unfolds.

More broadly, these findings highlight the value of feature-based representations for studying the perception–production interface in accented speech. Treating cue-specificity as a perception-grounded phenomenon provides a flexible framework for comparing accent patterns across speaker groups and contrasts, and for linking gradient production variability to perceptual outcomes in a transparent and theory-consistent manner.

## 7. Limitations and Future Directions

Several limitations of the present study point to important directions for future research. First, the analysis focused on two L1 backgrounds—Hindi and Tamil—that differ in their phonological feature inventories and patterns of feature co-occurrence. While this contrast allowed us to demonstrate language-specific pathways to perceived accentedness, future work should examine a broader range of language pairs to assess the generality of the feature-configuration account proposed here. In particular, extending this approach to L1s with different lateral systems, stop inventories, or vowel contrasts would help determine which aspects of feature-based accent perception are universal and which are language-specific.

Second, the study examined a limited set of segmental contrasts (laterals and labial segments) chosen for their theoretical relevance and cross-linguistic variability. Accentedness, however, emerges from the interaction of multiple segmental and suprasegmental cues. Future research should apply feature-based posterior representations to additional consonantal contrasts, vowel categories, and prosodic features to evaluate whether similar configuration-based perceptual mechanisms operate across domains.

Third, accentedness judgments in the present study were derived from native American English listeners using a coarse three-level rating scale. Because the CSLU FAE corpus is archival, the available accentedness ratings are limited to those provided in the corpus documentation (N = 3 raters), and additional perceptual data could not be collected for the present study. Although this design supports conservative statistical inference, finer-grained perceptual measures—such as continuous rating scales or paired-comparison tasks—may reveal more subtle relationships between phonological feature realization and perceived accent strength. Listener background and experience with different English varieties also merit systematic investigation. Controlled comparisons with accent ratings of native American English speech also merit inclusion for comparison.

Finally, while phonological posterior probabilities provide an interpretable and cross-linguistically grounded representation of speech, they are constrained by the feature inventory and training data used to build the model. Because CSLU FAE recordings are telephone-quality, channel limitations may attenuate fine-grained acoustic detail and could influence Phonet posterior probabilities; thus, feature posteriors should be interpreted as model-based acoustic evidence rather than direct articulatory measurements. In the present study, Phonet models were jointly trained on American English and accented English varieties, ensuring that phonological feature posteriors were derived from a shared representational space. This training regime allows differences in feature probabilities to be interpreted as reflecting variation in how speakers realize phonological features acoustically, rather than differences in how the model itself defines or weights those features.

Future work could contrast this joint-training approach with varietal-specific training, in which separate models are trained on individual language or accent varieties. Such an approach would allow the mapping between acoustic cues and phonological features to differ across varieties, making it possible to examine whether cue-specific drift reflects differences in which acoustic cues are most informative for signaling a feature in a given variety, rather than differences in production alone.

A further extension could also involve training feature-extraction models on a broader range of languages and accented varieties, allowing feature–cue relationships to be learned from more diverse phonetic systems. This multilingual training regime would provide a way to assess how stable and generalizable feature-based representations are across languages, and to identify which aspects of phonological feature realization are robust across linguistic systems versus language-specific.

In sum, despite these limitations, the present study demonstrates that feature-based representations offer a linguistically grounded framework for modeling how accentedness arises from gradient and cue-specific phonological organization. Extending this framework across languages, segments, and perceptual tasks represents a promising avenue for future research in L2 speech perception and production.

## Figures and Tables

**Table 1:** Comparison of English and Hindi consonant phonemes.

Place	English Consonants (IPA)	Hindi Consonants (IPA)
Bilabial	/p, b, m/	/p, p^h^, b, b^ɦ^, m/
Labiodental	/f, v/	/f, ʋ/
Dental	/θ, ð/	/t̪, d̪, t̪^h^, d̪^ɦ^/
Alveolar	/t, d, s, z, n, l, ɹ/	/s, z, n, l, ɹ/
Post-alveolar	/ʃ, ʒ, tʃ, dʒ/	/tʃ, tʃ^h^, dʒ, dʒ^h^/
Retroflex	—	/ʈ, ʈ^h^, ɖ, ɖ^ɦ^, ɻ, ɻ^ɦ^/
Palatal	/j/	/j/
Velar	/k, ɡ, ŋ/	/k, k^h^, ɡ, ɡ^ɦ^/
Glottal	/h/	/h/

**Table 2:** Comparison of English and Hindi vowel phonemes.

Vowel Type	English Vowels (IPA)	Hindi Vowels (IPA)
High Front	/i/, /ɪ/	/i/, /ɪ/
High Back	/u/, /ʊ/	/u/, /ʊ/
Mid Front	/e/, /ɛ/	/e/, /ɛ/
Mid central	—	/ə/
Mid Back	/o/, /ɔ/	/o/, /ɔ/
Low Front	/æ/, /a/	/æ/, /a/
Low Back	—	/ɑ/
Diphthongs	/aɪ/, /aʊ/, /ɔɪ/	/əɪ/, /əʊ/

**Table 3:** Comparison of Hindi and Tamil consonant phonemes.

Place	Hindi Consonants (IPA)	Tamil Consonants (IPA)
Bilabial	/p, p^h^, b, b^ɦ^, m/	/p, m/
Labiodental	/f, ʋ/	/ʋ/
Dental	/t̪, d̪, t̪^h^, d̪^ɦ^/	/t̪/
Alveolar	/s, z, n, l, ɹ/	/n, s, l, ɾ/
Post-alveolar	/tʃ, tʃ^h^, dʒ, dʒ^ɦ^/	/tʃ/
Retroflex	/ʈ, ʈ^h^, ɖ, ɖ^ɦ^, ɽ, ɽ^ɦ^/	/ʈ, ɳ, ʂ, ɻ̝, ɭ/
Palatal	/j/	/j, ɲ/
Velar	/k/	/k/
Glottal	/h/	---

**Table 4:** Comparison of Hindi and Tamil vowel phonemes.

Vowel Type	Hindi Vowels (IPA)	Tamil Vowels (IPA)
High Front	/i/, /ɪ/	/i:/, /ɪ/
High Back	/u/, /ʊ/	/u:/, /ʊ/
Mid Front	/e/, /ɛ/	/e:/, /e/
Mid central	/ə/	/ə/
Mid Back	/o/, /ɔ/	/o:/, /o/
Low Front	/æ/, /a/	—
Low Central	—	/ɐː/, /ɐ/
Low Back	/ɑ/	—
Diphthongs	/əɪ/, /əʊ/	—

**Table 5:** Comparison of English and Tamil consonant phonemes.

Place	English Consonants (IPA)	Tamil Consonants (IPA)
Bilabial	/p, b, m/	/p, m/
Labiodental	/f, v/	/ʋ/
Dental	/θ, ð/	/t̪/
Alveolar	/t, d, s, z, n, l, ɹ/	/n, s, l, ɾ/
Post-alveolar	/ʃ, ʒ, tʃ, dʒ/	/tʃ/
Retroflex	—	/ʈ, ɳ, ʂ, ɻ, ɭ/
Palatal	/j/	/j, ɲ/
Velar	/k, ɡ, ŋ/	/k/
Glottal	/h/	—

**Table 6:** Comparison of English vs. Tamil vowel phonemes.

Vowel Type	English Vowels (IPA)	Tamil Vowels (IPA)
High Front	/i/, /ɪ/	/i:/, /ɪ/
High Back	/u/, /ʊ/	/u:/, /ʊ/
Mid Front	/e/, /ɛ/	/e:/, /e/
Mid central	—	/ə/
Mid Back	/o/, /ɔ/	/o/, /ɔ/
Low Front	/æ/, /a/	—
Low Central	—	/ɐː/, /ɐ/
Diphthongs	/aɪ/, /aʊ/, /ɔɪ/	—

**Table 7:** Contrastive features among the lateral segments in American English, Hindi-accented English, and Tamil-accented English, as taken from [34].

Segment	[anterior]	[back]	[lateral]
/l/	+	0 *	+
/ɭ/	−	0	+
/ɻ/	−	0	−

* The ‘dark’ [ɫ] allophone of American English in word-final position is produced as a velarized consonant, with a sizeable gradient deviation in the [back] feature from the ‘light’ [l] allophone. Hindi- and Tamil-accented English lateral productions do not evince ‘dark’ allophones and the [back] feature is not expected to show large gradient variation. We follow the convention in [34] in marking [back] with zero feature values for lateral segments.

**Table 8:** Contrastive features among the /ʋ/, /v/ and /w/ segments in American English, Hindi English, and Tamil English, taken from [34]. We follow the convention in [34] of marking [dorsal] with zero feature values.

Segment	[consonantal]	[sonorant]	[approximant]	[round]	[labiodental]	[dorsal]
/ʋ/	−	+	+	−	+	0
/v/	+	−	−	−	+	0
/w/	−	+	+	+	−	+

**Table 9:** Pairwise feature probability differences by accent rating and word position, in segments produced by Hindi-accented English speakers. Only significant pairwise differences are reported following Bonferroni correction (*p* < 0.05). No significant interaction effects were observed.

Analysis	Feature	By Accent Rating ^1^	By Word Position ^2^
Laterals	[anterior]		I > M > F
	[back]	N > M; N > S	F > M > I
	[lateral]		I > M; I > F
Labiodental approximant	[approximant]	S > N	M > I
	[consonantal]	M > S	
	[sonorant]	M > N; S > N	M > I; M > F

^1^ N = no/negligible; M = mild; S = strong; ^2^ I = initial; M = medial; F = final.

**Table 10:** Pairwise feature probability differences by accent rating and word position and interactions between accent rating and word position, in segments produced by Tamil-accented English speakers. Only significant pairwise differences are reported following Bonferroni correction (*p* < 0.05).

Analysis	Feature	By Accent Rating ^1^	By Word Position ^2^	Interactions by Word Position ^1^	Interactions by Accent Rating ^2^
Laterals	[anterior]				I>F|mild; I>F, I>M|strong
	[lateral]			N>S, N>M|final	I>F, M>F|mild; I>F|strong
	[back]	N>M; N>S	F>M>I		
Labiodental approximant	[dorsal]	N>S			
	[high]	N>M; N>S			
	[back]	N>S			
	[tense]	N>S			

^1^ N = no/negligible; M = mild; S = strong; ^2^ I = initial; M = medial; F = final.

## Data Availability

All speech data used in this study are publicly available from the following sources: the CSLU Foreign Accented English (FAE) Corpus, Release 1.2 (Ref. [33]) CSLU: Foreign Accented English Release 1.2—Linguistic Data Consortium (https://catalog.ldc.upenn.edu/LDC2007S08, accessed on 15 July 2025); the Mozilla Common Voice Dataset (Ref. [35]) Common Voice: A Massively-Multilingual Speech Corpus—ACL Anthology (https://aclanthology.org/2020.lrec-1.520/, accessed on 1 July 2025); the LibriSpeech ASR Corpus (train-clean-100 subset, accessed on 1 July 2025) (Ref. [36]) openslr.org (https://www.openslr.org/12, accessed on 1 July 2025); the L2-ARCTIC Corpus (Ref. [37]) L2-ARCTIC: a non-native English speech corpus—PSI Lab (https://psi.engr.tamu.edu/l2-arctic-corpus/, accessed on 15 July 2025); the Indic Text-to-Speech (IndicTTS) Corpus (Ref. [38]) GitHub version 3.5.4; and AI4Bharat/Indic-TTS: Text-to-Speech for languages of India (https://github.com/AI4Bharat/Indic-TTS, accessed on 23 July 2025); the IndicTIMIT Corpus (Ref. [39]) IndicTIMIT CORPUS (https://spiredatasets.ee.iisc.ac.in/indictimitcorpus?utm_, accessed on 23 July 2025).

## Appendix A

Table A1 and Table A2 show the phonological feature classification accuracy and F1 scores of the two Phonet models trained respectively on the American/Hindi English and American/Tamil English pairs.

**Table A1:** Phonological feature classification scores for the Phonet model trained on American English and Hindi English data.

Feature	Accuracy	F1-Score
Anterior	93.31	93.67
Approximant	95.18	95.20
Back	92.03	93.48
Consonantal	93.99	94.02
Dorsal	94.01	94.03
High	91.95	92.45
Labiodental	95.77	96.38
Lateral	96.39	97.01
Round	95.18	95.69
Sonorant	97.43	97.55
Tense	88.45	89.46

**Table A2:** Phonological feature classification scores for the Phonet model trained on American English and Tamil English data.

Feature	Accuracy	F1-Score
Anterior	93.72	94.07
Approximant	95.42	95.45
Back	92.23	93.66
Consonantal	94.18	94.22
Dorsal	94.4	94.44
High	91.71	92.3
Labiodental	96.18	96.72
Lateral	97.12	97.57
Round	95.49	95.95
Sonorant	95.69	95.7
Tense	89.65	90.61

**Table A3:** Mapping between MFA phonesets and Hayes’ phonological classes for Phonet modeling. Phonesets are taken from the MFA English dictionaries for American English, Hindi English and Tamil English.

Phonological Class	Phone List
syllabic	a aj aw a: e: ej i i: o: ow æ ɐ ɑ ɑː ɒ ɒː ɔj ə ɚ ɛ ɛː ɜ ɜː ɝ ɪ ʉ ʉː ʊ
consonantal	b b^j^ c c^h^ c^w^ d dʒ d^j^ d̪ f f^j^ h j k k^h^ k^w^ l m m^j^ m̩ n n̩ p p^h^ p^j^ p^w^ s t tʃ t^h^ t^j^ t^w^ t̪ v v^j^ z ç ð ŋ ɖ ɟ ɟ^w^ ɡ ɡ^w^ ɫ ɫ̩ ɱ ɲ ɾ ɾ^j^ ɾ̃ ʃ ʈ ʈ^j^ ʈ^w^ ʎ ʒ ʋ ʔ θ
long	a: e: i: o: ɑː ɒː ɛː ɜː ʉː
sonorant	a aj aw a: e: ej i i: o: ow æ ɐ ɑ ɑː ɒ ɒː ɔj ə ɚ ɛ ɛː ɜ ɜː ɝ ɪ ʉ ʉː ʊ l ɫ ɫ̩ ʎ j ɾ ɾ^j^ ɾ̃ m m^j^ m̩ ɱ n n̩ ɲ ɹ ŋ ʋ w
continuant	a aj aw a: e: ej i i: o: ow æ ɐ ɑ ɑː ɒ ɒː ɔj ə ɚ ɛ ɛː ɜ ɜː ɝ ɪ ʉ ʉː ʊ ð θ j ɾ ɾ^j^ ɾ̃ ʃ ʒ ʋ ɹ v v^j^ z ç ʎ v v^j^ s h w l ɫ ɫ̩
approximant	a aj aw a: e: ej i i: o: ow æ ɐ ɑ ɑː ɒ ɒː ɔj ə ɚ ɛ ɛː ɜ ɜː ɝ ɪ ʉ ʉː ʊ j ɾ ɾ^j^ ɾ̃ l ɫ ɫ̩ ʎ ʋ w ɹ
delayed release	f f^j^ ʃ ʒ v v^j^ ç dʒ tʃ h s z ð θ
tap	ɾ ɾ^j^ ɾ̃
nasal	m m^j^ m̩ ɱ n n̩ ɲ ŋ
voice	a aj aw a: e: ej i i: o: ow æ ɐ ɑ ɑː ɒ ɒː ɔj ə ɚ ɛ ɛː ɜ ɜː ɝ ɪ ʉ ʉː ʊ b b^j^ d dʒ d^j^ ð d̪ ɖ ɾ ɾ^j^ ɾ̃ l ɫ ɫ̩ j ɹ ʒ v v^j^ z m m^j^ m̩ ɱ n n̩ ɲ ŋ ʎ ɡ ɡ^w^ ʋ w
spread glottis	h
labial	p p^h^ p^j^ p^w^ f f^j^ v v^j^ ʋ m m^j^ m̩ ɱ b b^j^
round	ɒ ɒː o: ow ʉ ʉː ʊ ɔj
dental	t̪ d̪ ð θ
coronal	c c^h^ c^w^ ç ɾ ɾ^j^ ɾ̃ ɹ dʒ ʃ ʒ tʃ ʈ ʈ^j^ ʈ^w^ t t^h^ t^j^ t^w^ t̪ n n̩ ɲ ʎ d d^j^ d̪ ɖ l ɫ ɫ̩ s z ð θ
anterior	ɾ ɾ^j^ ɾ̃ t t^h^ t^j^ t^w^ t̪ d d^j^ d̪ n n̩ l ɫ ɫ̩ s z ð θ
distributed	c c^h^ c^w^ ç ɹ dʒ ʃ ʒ ɟ ɟ^w^ tʃ ɲ ʎ ð θ
strident	s z dʒ ʃ ʒ tʃ
lateral	l ɫ ɫ̩ ʎ
dorsal	a aj aw a: e: ej i i: o: ow æ ɐ ɑ ɑː ɒ ɒː ɔj ə ɚ ɛ ɛː ɜ ɜː ɝ ɪ ʉ ʉː ʊ c c^h^ c^w^ ç ɡ ɡ^w^ k k^w^ ʎ ɲ ŋ w
high	i i: ɪ ʉ ʉː ʊ c c^h^ c^w^ ç ɡ ɡ^w^ k k^w^ ʎ w ɲ ŋ
low	a aj aw a: ɑ ɑː ɒ ɒː æ
front	æ ɛ ɛː i i: ɪ e: ej c c^h^ c^w^ ç j ʎ ɲ ɟ ɟ^w^
back	ɑ ɑː ɒ ɒː ɜ ɜː ɝ o: ow ɔj ʊ ɫ ɫ̩ w
tense	e: ej i i: ʉ ʉː o: ow ə ɚ j w
constr. glottis	ʔ
